# *Campylobacter fetus* foodborne illness outbreak in the elderly

**DOI:** 10.3389/fmicb.2023.1194243

**Published:** 2023-07-06

**Authors:** Gaspard Grouteau, Cédric Mignonat, Bruno Marchou, Guillaume Martin-Blondel, Olivier Glass, Claire Roubaud-Baudron, Pauline Lansalot-Matras, Simon Alik, Laurent Balardy, Thomas De Nadaï, Lucie Bénéjat, Quentin Jehanne, Alain Le Coustumier, Philippe Lehours

**Affiliations:** ^1^Infectious and Tropical Diseases Department, Centre Hospitalier Tarbes-Lourdes, Lourdes, France; ^2^Rehabilitation Center, L'Arbizon, Bagnères de Bigorre, France; ^3^Infectious and Tropical Diseases Department, CHU de Toulouse, Toulouse, France; ^4^Institut Toulousain des Maladies Infectieuses et Inflammatoires (Infinity) INSERM UMR1291—CNRS UMR5051—Université Toulouse III, Toulouse, France; ^5^Cellule de veille d'alerte et de gestion sanitaire, Agence Régionale de Santé Occitanie, Toulouse, France; ^6^Pôle de Gérontologie Clinique, CHU de Bordeaux, Bordeaux, France; ^7^Bordeaux Institute of Oncology, BRIC U1312, INSERM, Université de Bordeaux, CHU de Bordeaux, Bordeaux, France; ^8^Gerontopole of Toulouse, CHU de Toulouse, Toulouse, France; ^9^National Reference Center for Campylobacters and Helicobacters, Bacteriology Department, CHU de Bordeaux, Bordeaux, France; ^10^Bacteriology Department, Centre Hospitalier Tarbes-Lourdes, Tarbes, France

**Keywords:** *Campylobacter fetus*, bacteremia, food safety, foodborne outbreak, foodborne illness, elderly, outbreak, WGS analysis

## Abstract

In June 2021, a cluster of seven cases of *Campylobacter fetus* infections occurred in a rehabilitation center and caused significant morbidity in elderly patients including five with bacteremia and two with osteoarticular medical device infections. The genetic identity identified by whole genome sequencing of the different *Campylobacter fetus* strains confirms a common source. This foodborne illness outbreak may have resulted from the consumption of unpasteurized dairy products, such as a cow's raw milk cheese resulting from a farm-to-fork strategy.

## Introduction

*Campylobacter* spp. are small, curved, Gram-negative rods. *Campylobacter jejuni* and *Campylobacter coli* are established pathogens in human acute gastroenteritis and cause the majority of intestinal campylobacteriosis, and a small proportion is caused by *Campylobacter fetus* (*C. fetus*; Gazaigne et al., 2008; Man, 2011). Extra-digestive manifestations such as bacteremia due to *Campylobacter* spp. are uncommon, with an incidence of 2.9 per 1 million person-years described in Denmark and affecting mainly subjects older than 80 years (Nielsen et al., 2010). Although *C. fetus* may occasionally cause diarrhea, it has been more associated with systemic illness and bacteremia with possible extra-digestive localizations including endovascular infections, bone and joint infection, and cellulitis (Gazaigne et al., 2008; Pacanowski et al., 2008; Wagenaar et al., 2014; Tinévez et al., 2022). *C. fetus* infections are more commonly reported in older and immunocompromised patients (Pacanowski et al., 2008; Wagenaar et al., 2014; Tinévez et al., 2022). In addition, foreign medical device implants also appear to be a risk factor for *C. fetus* infections, such as prosthetic valve endocarditis, vascular graft infections, and periprosthetic joint infections (Tinévez et al., 2022).

*C. fetus* has a surface layer (S-layer), a paracrystalline lattice that coats the outer membrane of these bacteria, actually identified as the major virulence factor, explaining its extra-intestinal spread (Man, 2011). The S-layer disrupts C3b binding to these bacteria, explaining both serum and phagocytosis resistance (Man, 2011). *C. fetus* is an accidental pathogen of humans, the combination of its immune avoidance and the presence of host predispositions can be sufficient to cause infection (Wagenaar et al., 2014).

*C. fetus* outbreaks are infrequent: six have been potentially linked to contaminated food exposure (Taylor et al., 1979; Itoh et al., 1980; Klein et al., 1986; Rennie et al., 1994; Wagenaar et al., 2014) and two have been linked to possible person-to-person transmission (Morooka and Takeo, 1988; Marchand-Senécal et al., 2017). Food products from cattle and sheep such as raw milk, unpasteurized dairy products, raw liver, and raw meat seem to be the main source of human infection (Wagenaar et al., 2014). Such clusters among the elderly have been reported from 1981 to 1993 and were not completely characterized (Wagenaar et al., 2014).

The present study describes a *C. fetus* cluster among elderly patients in a rehabilitation center (RC) located in southwestern France. The aim was to describe and investigate this outbreak in order to better understand the transmission and raise awareness of this infectious disease among physicians in charge of at-risk patients such as the elderly or immunocompromised patients. A clinical case description and an epidemiological and microbiological investigation were made to link the cases and try to find a common source of infection.

## Methods

A descriptive retrospective study of patients who presented with a *C. fetus* infection in an RC located in South-West France was conducted in June 2021. Following French legislation, relating to outbreaks of foodborne illness (decree n° 2001-671 of 26 July 2001), the French Institute of Public Health (Agence Régionale de Santé, Occitanie, France) was notified of the occurrence of a cluster of febrile enteritis among hospitalized patients. All patients were informed of this procedure. Data were pseudonymized, the included patients who were still alive did not object to the analysis of their data for research purpose and publication. Furthermore, patients' medical records were reviewed in order to gather information about sociodemographic data, comorbidities, clinical symptoms, secondary localizations, treatment, and outcomes.

### Microbiological investigation

All samples of patients showing symptoms (blood culture, stools, and synovial fluid) were sent to the National Reference Center (NRC) for *Campylobacter* in Bordeaux, France, for strain identification using matrix-assisted laser desorption ionization time-of-flight mass spectrometry and for determining antimicrobial susceptibility to ampicillin, amoxicillin-clavulanate, ciprofloxacin, erythromycin, tetracycline, gentamicin, and imipenem using disk diffusion according to EUCAST guidelines (http://www.eucast.org). Each blood culture set (aerobic and anaerobic bottle) was inoculated with 10 ml of blood, incubated for 5 days, and continually monitored using non-invasive blood culture systems Bactec^TM^, Becton Dickinson. Blood from a positive blood culture bottle and synovial fluid was inoculated in a blood agar plate. Stool samples were inoculated in a *Campylobacter*-selective media for isolation by culture. All samples were incubated in a microaerobic atmosphere at 35°C for 48 h.

A specific nucleic acid amplification test with real-time fluorescence resonance energy transfer (FRET) PCR was performed to detect *C. fetus* in stool and synovial fluid that were found to be negative by culture only (Ménard et al., 2005).

Other enteric pathogens have been investigated in all stool samples. A multiplex real-time PCR gastroenteric bacterial (I) assay, Allplex^TM^ (Seegene) was used to detect enteric bacterial agents: *Salmonella* spp., *Campylobacter* spp., *Shigella* spp., *E. coli* EIEC, *Yersinia enterocolitica, Aeromonas* spp., and *Vibrio* spp. Extraction and amplification were, respectively, performed with Hamilton Starlet/Nimbus^TM^ and Biorad CFX96^TM^ systems. For viral agent detection, stool samples were sent to the NRC for gastroenteritis virus in Dijon, France. TaqMan^TM^ fast Virus 1-step, Applied Biosystems real-time PCR was used for *Astrovirus, Norovirus, Rotavirus*, and *Sapovirus* detection. ADENOVIRUS R-gene^TM^, BioMérieux real-time PCR was used for *Adenovirus* detection.

### Genetic investigation

Pure DNA samples from each *C. fetus* isolate were obtained from bacterial growth using MagNA Pure 6 DNA and viral NA SV Kit (MagNA Pure 96 system, Roche Applied Science, Manheim, Germany). Paired-end next-generation sequencing (NGS) was performed using Illumina Nextera DNA Library Preparation Kit and Iseq 100 system (300-cycles reagent, 2 × 150 bp reads generation). FastQC v0.11.9 (Wingett and Andrews, 2018) was used to run data quality tests, and reads were cleaned and assembled using Sickle v1.33 (Joshi and Fass, 2011) and SPAdes v3.15.5 *de novo* methods (Bankevich et al., 2012), respectively. Species were identified using the Average Nucleotide Identity (ANI) algorithm of FastANI v1.1 (Jain et al., 2018) against 68 various reference species of *Campylobacter* (including *C. fetus* CFF00A031, *C. fetus subsp. fetus* NCTC 10842, C. *fetus subsp. testudinum* pet-3, *C. fetus subsp. fetus* CCUG 6823, and *C. fetus subsp. venerealis* NCTC 10354). Whole-genome SNP phylogeny was performed from the alignment of each *C. fetus* isolate combined with 75 additional clinical isolates (Iraola et al., 2017) against *C. fetus* reference CFF00A031 using *bwa mem* v0.7.17 (Li, 2013) and SAMtools v1.13 (Li, 2011). Specifically, pairwise comparisons of each variable position within the genome were performed in order to compute distances between all isolates: a score of 100% between two isolates means they share the same set of SNPs. Scores were finally displayed in a phylogenetic tree using iTOL online tool v6 (Letunic and Bork, 2021). Additionally, resistance and virulence genes were identified using Blast 2.12.0+ command line tool (Camacho et al., 2009) as well as the following databases: NCBI, CARD, ResFinder, VFDB, and VirulenceFinder, and 150 *C. fetus* proteins from UniProt have been described as responsible for the bacteria virulence.

### Epidemiological investigation

An environmental investigation was performed by the French public health agency with traceback investigation including the inspection of RC kitchens. The date of onset of symptoms and the details of the food consumed at each meal by symptomatic patients in the 4 days preceding the manifestations of symptoms were collected. Details of food consumed by confirmed cases were analyzed. Hypotheses were then made to find the probable source of infection.

## Results

From 2 June 2021 to 21 June 2021, seven consecutive cases of *C. fetus* infections were identified from a single RC. All the patients were female, and the median age was 79 years [range 70–90 years] (Table 1). The reasons for RC admission were post-orthopedic surgery reeducation (5/7), post-acute heart failure (1/7), and rehabilitation after acute myeloid leukemia chemotherapy (1/7). The functional status assessed by activities of daily living (ADL) score was altered in 4 out of 7 cases, median ADL was 3.5 (IQR 3–6), and one patient lived in a long-term care facility.

**Table 1:** Summary of the demographic data, clinical, and microbiological characteristics for the seven cases of *C. fetus* infection.

**Cases**	**Sex/ age**	**Underlying condition**	**Clinical signs**	**Origin of *C. fetus* isolates**	**NGS sequencing of the strain/origin**	**Secondary localization**	**Outcomes**
**#**1	F/78	Cardiopathy, polymedication^a^ ≥ 5, shoulder prosthesis, pacemaker	Fever, diarrhea, prosthetic shoulder pain	Blood culture^b^, stools^b*^	Yes/blood culture Genome ID^**^: 1	Shoulder Prosthesis	Favorable, 12w clarithromycin after amoxicillin
**#**2	F/88	Breast cancer, ADL 3.5/6, polymedication^a^, diabetes, hip replacement, pacemaker	Fever, diarrhea, prosthetic hip collection	Blood culture^b^, hip collection^c^ and stools^c^	Yes/blood culture Genome ID^**^: 2	Hip prosthesis	Favorable, 12w ciprofloxacin after amoxicillin and surgical debridement
**#**3	F/79	ADL 3/6, hip replacement, polymedication^a^, penicillin allergy	Fever, diarrhea	Blood culture^b^ with *Listeria* co-infection, stools^c^	Yes/blood culture Genome ID^**^: 3	None	Favorable with ciprofloxacin + Sulfamethoxazole-Trimethoprim
**#**4	F/77	ADL 6/6, cervical arthrodesis	Fever, diarrhea	Blood culture^b^, stools^d^	Yes/2 strains: blood culture and stools Genome ID^**^: 4A, 4B	None	Favorable without antibiotic
**#**5	F/90	ADL 6/6, Hip Zimmer Natural Nail, aortic prosthesis	Diarrhea without fever	Stools^c^	No	None	Favorable without antibiotic
**#**6	F/70	ADL 3/6, Hematologic cancer with chemotherapy, diabetes	Diarrhea without fever	Blood culture^b^	Yes/2 strains from blood culture taken at different times Genome ID^**^: 6A, 6B	None	Favorable, 1w ciprofloxacin after amoxicillin
**#**7	F/84	ADL 6/6, knee and hip replacement	Fever, diarrhea	Stools^c^	No	None	Favorable without antibiotic

Age is indicated in year.

ADL, Activities of Daily Living; F, female; max. T°C, maximal temperature degree Celsius; NGS, next-generation sequencing; w, week.

^a^Polymedication ≥ 5 long-term treatment.

^b^Classic bacteriological culture.

^c^Detection of *C. fetus* only by specific PCR.

^d^Detection by specific PCR and culture.

^*^This *C. fetus* isolate was not sequenced.

**Figure 1:**
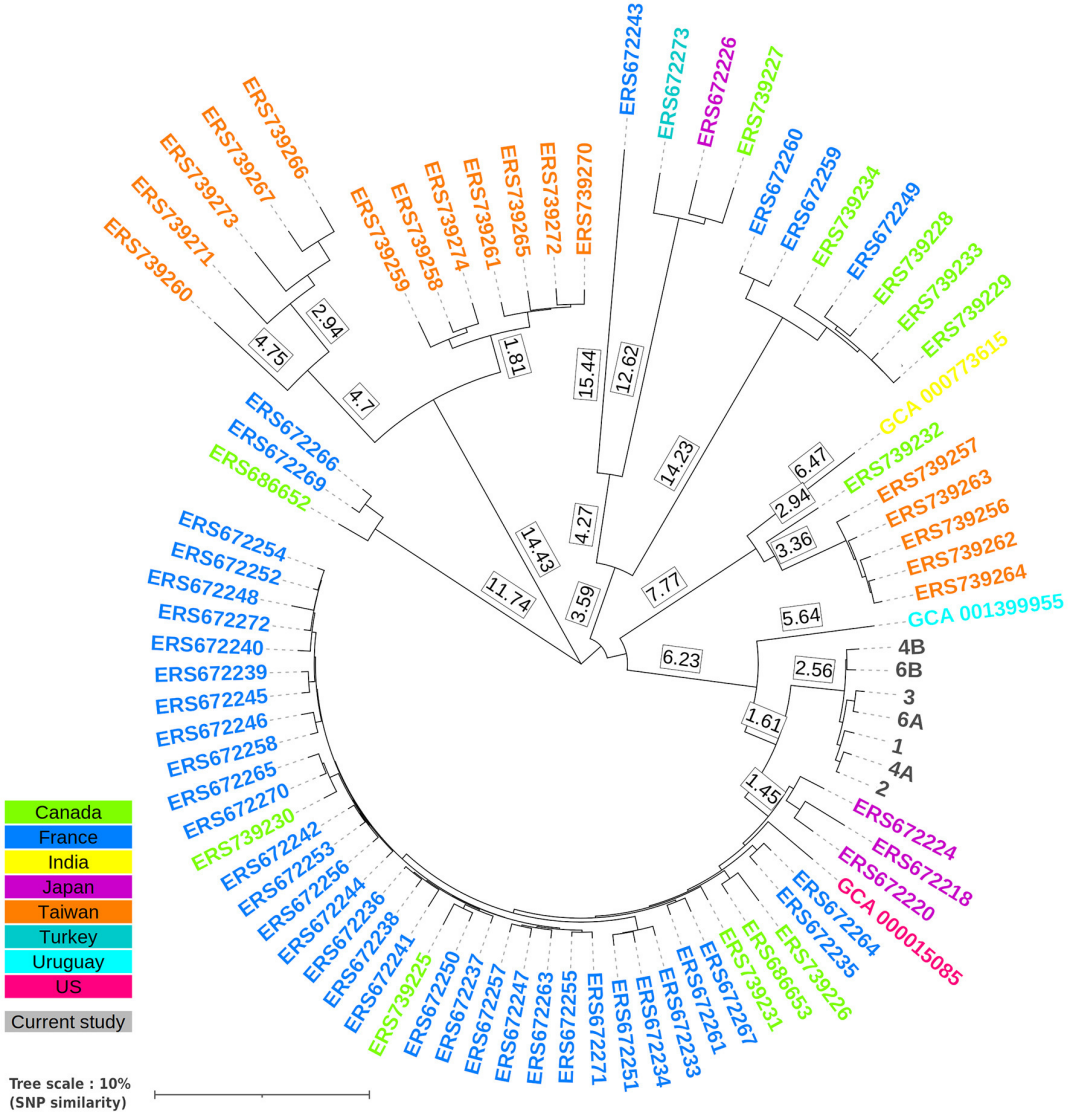
Whole-genome SNP phylogenetic tree generated from the alignment of each *C. fetus* studied isolate (cases 1, 2, 3, 4A, 4B, 6A, and 6B) combined with 75 additional clinical isolates (Iraola et al., 2017) from various countries against *C. fetus* reference CFF00A031. Genomes were aligned against the reference using *bwa mem*, and SNPs were called using *samtools*. Pair-wise comparison between all genomes was performed in order to determine the proportion of genotype similarity, which is displayed in the present tree in the percentage of identical SNP using iTOL online tool v6.

^**^Genome ID: Genome identity of *C. fetus* strain as shown in Figure 1.

All patients presented mild watery diarrhea with a maximum of 5 stools per day for 5 days, without nausea or vomiting. Five of them had fever with an average maximum temperature of 38.5°C (± 0.41); none developed sepsis. Two patients out of the six with osteoarticular implanted medical devices had infections, respectively, of their hip and shoulder prosthesis, which were confirmed using a CT scan for the first and fluorodeoxyglucose positron emission tomography (PET-CT) for the second (Table 1, case #1, #2). A CT scan was performed in the case of two patients, transthoracic echocardiography in the case of two patients, ultrasonography with one patient, and a PET-CT scan with one patient. Biological inflammation markers showed a median C-reactive protein level of 165 (IQR 112–235) mg/L and a median leucocyte count of 12.9 (IQR 8.3–18.9) G/L.

In five patients who undertook a blood culture analysis, *C. fetus* was isolated only in the blood of three patients and was accordingly isolated from blood and stool in two of them (Table 1). *C. fetus* was detected by specific PCR in six patients including the two patients in whom *C. fetus* was only detected in the stools by this method (Table 1, cases #5, #7). *C. fetus* was also detected using specific PCR performed on synovial fluid harvested during surgical debridement which occurred after antibiotic treatment was initiated in one patient (Table 1, case #2).

No other enteric pathogen was found in the stools, but one patient had concomitant *Listeria monocytogenes* bacteremia (Table 1, case #3). All *C. fetus* isolates were susceptible to ampicillin, amoxicillin + clavulanic acid, gentamicin, erythromycin, ciprofloxacin, and tetracycline.

All patients had favorable outcomes. Of the two patients with prosthetic osteoarticular infections, only one had surgical debridement, and both received a 12-week course of antibiotic treatment. Three patients with symptoms of gastroenteritis recovered without antibiotic treatment including one with concomitant *C. fetus* bacteremia (Table 1, case#4).

The complete strain identification revealed a *C. fetus* subspecies *fetus*. Whole genome sequencing by NGS of the seven *C. fetus* isolates, collected confirmed a genetic identity matching for that specific species. ANI scores were, in fact, highly significant against *C. fetus* CFF00A031 reference, from 99.986 to 99.996% (as a reminder, ANI positive cutoff needs to be greater or equal to 95%). Despite the lack of data for *Campylobacter* spp. clonality identification, especially SNP rate thresholds, phylogeny based on wgSNP analysis revealed a distinct cluster composed of seven studied isolates (Figure 1). Precisely, only 119 variable positions were found between all 7 isolates, representing an average percentage of whole-genome similarity of ~98.79% ± 0.37, suggesting a strong genomic relationship between each clinical case. Although no genome displayed any known resistance marker, 37 virulence proteins were found among each isolate, highlighting their putative virulence. A list of these proteins with their associated sequences is presented in Supplementary Table 1. It includes three sets of CdtA, B, and C subunits (two out of three located next to each other), seven ABC transporters or adhesin-related proteins, two S-layer proteins, three chemotaxis proteins, and 12 flagellar or motility-related proteins (PseB, FlhA, FliP, FliQ, FlgC, FlgG, FliG, FliN, FliM, FlgP, FlgR, and FliL). Furthermore, all protein sequences are fully identical between all studied isolates. The seven different strains came from five patients. Specifically, two isolates for one patient were sampled from both stools and blood, two other isolates were sampled from blood from the same patient, and the remaining three isolates were sampled from blood from three distinct patients. Raw sequencing data of each sample are available under ENA Study accession number PRJEB62826 and ERS15567209 for case #1 isolate, ERS15567208 for case #2 isolate, ERS15567207 for case #3 isolate, ERS15567203 for isolate 1 of case #4, ERS15567204 for isolate 2 of case #4, ERS15567205 for isolate 1 of case #6, and ERS15567206 for isolate 2 of case #6.

Epidemiological investigations found three simultaneous cases on 2 June followed by four cases on 5, 7, 18, and 20 June 2021. An investigation of the food consumed by five patients revealed the consumption of unpasteurized cheese made from cow milk, a “Tomme des Pyrénées,” 2 and 3 days before the three simultaneous first cases, 5 and 6 days before the fourth case, and 7 and 8 days before the fifth case. Another unpasteurized cheese made from cow milk, a “Camembert,” was also consumed the day before the three first cases, 4 days before the fourth case, and 6 days before the fifth case. These two unpasteurized milk cheeses were issued from a recently implemented farm-to-fork strategy. No undercooked meat was consumed. Out of 70 patients who were potentially exposed at the same time in the RC to the same contaminated food item, seven *C. fetus* infections were recorded. A traceback was performed by French public health authorities, which revealed serious hygiene issues in the kitchens, with disrespect of “cold-chain” guidelines leading to their closure on 14 June 2021. Unfortunately, no food testing investigation was made. Farm-to-fork strategies were pursued but unpasteurized dairy products were avoided. No other case was found after 21 June 2021.

## Discussion

Few *C. fetus* outbreaks have been reported in the literature and they rarely involved the elderly (Klein et al., 1986; Rennie et al., 1994; Wagenaar et al., 2014). Evidence suggests that contaminated food, especially products made up of raw milk and undercooked meat may be the sources of human *C. fetus* infection. This evidence comes from epidemiological investigations of outbreaks and sporadic *C. fetus* illnesses (Wagenaar et al., 2014). Our study provides elements in favor of these assumptions with an outbreak of seven *C. fetus* enteritis with three concomitant initial cases, following the consumption of unpasteurized cheese made from cow milk. Raw milk cheeses were mainly suspected due to the fact that there was no consumption of other plausible sources of infection such as undercooked meat. The incubation period of campylobacteriosis varies from a few hours to 14 days, and the mean incubation period ranges from 2.4 to 4.3 days (Awofisayo-Okuyelu et al., 2017). This incubation period was consistent with the hypothesis that the source of infection was the raw milk cheese. However, it was not possible to clearly incriminate one of the two different raw milk cheeses, the “Tomme des Pyrénées” or “Camembert.” The genetic identity of the seven different strains confirms a common source. Unfortunately, the source of the *C. fetus* infection was not proven because of the absence of microbiological analysis of the suspected food samples. The last two cases occurred 16 and 18 days later, suggesting either the persistence of the food source or interhuman transmission (Itoh et al., 1980; Marchand-Senécal et al., 2017). Concomitant *Listeria monocytogenes* bacteremia in one patient also raises the suspicion that raw milk cheese was the source of the infection. This fact also clearly demonstrates how susceptible the elderly or immunocompromised patients are to this type of foodborne infection.

This outbreak confirmed the invasive potential of *C. fetus*, especially in the elderly and immunocompromised host. *C. fetus* infections have usually been associated with invasive infections such as bacteremia with possible secondary localization (Pacanowski et al., 2008; Wagenaar et al., 2014; Tinévez et al., 2022). In a recent study describing 252 *C. fetus* bacteremia cases, patients were older than those with *C. jejuni* bacteremia cases, and secondary localizations were found in 24.2% of cases, mainly with endovascular infections (11.5%) followed by osteoarticular infections (7.2%; Tinévez et al., 2022). While diarrhea is inconstantly associated with systemic infections (Pacanowski et al., 2008; Tinévez et al., 2022), all the patients reported here had mild diarrhea, and *C. fetus* was found in six out of seven stool samples. The occurrence of a cluster of foodborne illnesses may have alerted the clinicians to look for even mild digestive symptoms in all patients. In sporadic cases, possible digestive symptoms preceding a systemic infection with *C. fetus* may have gone unnoticed. The median age of the patient is also a possible risk factor for digestive symptoms. Possible exposure to antibiotics before the outbreak was not described.

The optimal treatment of *C. fetus* infections is not well-defined, and to our knowledge, there are no randomized controlled trials to evaluate it. Several studies conducted in Quebec and France found preserved susceptibility to amoxicillin-clavulanate, imipenem, meropenem, and gentamicin (Tremblay et al., 2003; Gazaigne et al., 2008; Pacanowski et al., 2008; Tinévez et al., 2022). Ampicillin resistance was recently described at 7.2% in a French study (Tinévez et al., 2022). Ciprofloxacin resistance was found to be at 3% in Quebec in 2003 and at 30% in France in 2022 (Tremblay et al., 2003; Tinévez et al., 2022). Resistance to tetracycline and erythromycin was observed in 15 and 1.7% of *C. fetus* isolates, respectively, in the same French study (Tinévez et al., 2022). Cefotaxime was described to have a lower *in vitro* bactericidal activity compared with ampicillin, gentamicin, and imipenem, and its susceptibility was described as intermediate in 12–62% of studied strains in Canada and France, respectively (Spelhaug et al., 1981; Tremblay et al., 2003; Gazaigne et al., 2008). The use of third-generation cephalosporins was also described to be a risk factor for death in *Campylobacter* spp. bacteremia with species other than *C. fetus* (Pacanowski et al., 2008). In our study, all strains remained susceptible to ampicillin, amoxicillin-clavulanate, ciprofloxacin, erythromycin, tetracycline, gentamicin, and imipenem. Different antibiotic regiments were used, and in three patients, no antibiotics were taken including one with *C. fetus* bacteremia (Table 1). This fact confirmed the possibility of transient *C. fetus* bacteremia, resolved without antimicrobial therapy. However, failure to administer appropriate antibiotics is strongly associated with fatal outcomes in *Campylobacter* spp. bacteremia (Pacanowski et al., 2008).

This study had several limitations in addition to being retrospective and also having only a small number of subjects. First, a cohort study would have been appropriate to calculate an attack rate and relative risks, according to different food samples. Second, a systematic evaluation of asymptomatic residents exposed during this period was not performed, leading to a possible underestimation of *C. fetus* infection. Third, the epidemiological data hypothesized unpasteurized milk cheese to be the source of infection, but microbiological analysis of food samples was not performed.

## Conclusion

In conclusion, we report a cluster of *C. fetus* infections with diverse clinical patterns in elderly people with a plausible common food source. This highlights the importance of routine hygienic measures to prevent transmission within facilities housing people at risk. Even if the source of infection was only suspected and not confirmed, consumption of unpasteurized dairy products should also be avoided in these specific populations.

## Data availability statement

Original datasets are available in the Supplementary material and have been deposited in a publicly accessible repository. Raw sequencing data of each sample are available under ENA Study accession number: PRJEB62826 and ERS15567209 for case 1 isolate, ERS15567208 for case 2 isolate, ERS15567207 for case 3 isolate, ERS15567203 for isolate 1 of case 4, ERS15567204 for isolate 2 of case 4, ERS15567205 for isolate 1 of case 6 and ERS15567206 for isolate 2 of case 6. Further inquiries can be directed to the corresponding authors.

## Ethics statement

Ethical review and approval was not required for the study on human participants in accordance with the local legislation and institutional requirements. Written informed consent for participation was not required for this study in accordance with the national legislation and the institutional requirements. Written informed consent was not obtained from the individual(s) for the publication of any potentially identifiable images or data included in this article.

## Author contributions

GG, CM, and BM contributed to the clinical follow-up and patient interviews. OG contributed to the epidemiological investigation. GG, CM, PL-M, SA, and AL collected the patients' data. LBé, QJ, and PL contributed to the laboratory investigations in the Campylobacter National Reference Center based in Bordeaux. GG, BM, GM-B, CR-B, and PL wrote the first draft of the manuscript. LBa and TD contributed to the revision of the manuscript. PL supervised the project. All authors contributed to the study conception, commented on previous versions of the manuscript, read, and approved the final manuscript.
